# CD4^+^ T cell exhaustion revealed by high PD-1 and LAG-3 expression and the loss of helper T cell function in chronic hepatitis B

**DOI:** 10.1186/s12865-019-0309-9

**Published:** 2019-08-07

**Authors:** Yuejiao Dong, Xuefen Li, Lu Zhang, Qiaoyun Zhu, Chunlei Chen, Jiaqi Bao, Yu Chen

**Affiliations:** 1 Department of Laboratory Medicine, Key Laboratory of Clinical In Vitro Diagnostic Techniques of Zhejiang Province, First Affiliated Hospital, College of Medicine, Zhejiang University, 79 Qingchun Road, Hangzhou, 310003 China; 2 Collaborative Innovation Center for Diagnosis and Treatment of Infectious Diseases, State Key Laboratory for Diagnosis and Treatment of Infectious Diseases, The First Affiliated Hospital, College of Medicine, Zhejiang University, 79 Qingchun Road, Hangzhou, 310003 China

**Keywords:** CD4^+^ T cells, Chronic HBV infection, Inhibitory molecules, Programmed death 1 (PD-1), Lymphocyte activation gene-3 (LAG-3), Cytokine

## Abstract

**Background:**

Immune inhibitory receptors play an important role in chronic infections. However, little is known about their role in hepatitis B virus (HBV) infection. Here, we analyzed the relationship between programmed death-1 (PD-1) and lymphocyte activation gene-3 (LAG-3) expression on CD4^+^ T cells and HBV disease progression.

**Results:**

PD-1 and LAG-3 expression was significantly higher on CD4^+^ T cells from HBV patients than on those from the HCs. In addition, a significant positive correlation was found between the PD-1 and LAG-3 expression levels and the ALT(alanine aminotransferase) level. CD4^+^ T cell function was inhibited by high PD-1 and LAG-3 levels, and CD4^+^ T cells with high PD-1 and LAG-3 expression lost the ability to secrete IFN-γ, IL-2 and TNF-α. Furthermore, blockade of the PD-1 and LAG-3 pathways reversed the damage to CD4^+^ T cell proliferation and cytokine secretion.

**Conclusions:**

CD4^+^ T cell exhaustion during chronic HBV had high PD-1 and LAG-3 expression and the absence of helper T cell cytokines, including IFN-γ, IL-2 and TNF-α. After blocking PD-L1 and LAG-3, CD4^+^ T cell function in chronic hepatitis B patients was partially restored.

## Background

Chronic hepatitis B virus (HBV) infection is a serious public health challenge that can result in severe consequences, such as liver cirrhosis and hepatocellular carcinoma [1]. Vigorous immune responses against HBV, including HBV-specific T cell and helper T cell responses, are thought to play a dominant role in viral clearance and disease pathogenesis as well as in preventing or reducing the prevalence of liver cirrhosis and liver cancer [2, 3]. During virus infection, CD8^+^ T cells urgently require CD4^+^ T cells because CD8^+^ T cell functions are seriously damaged and are gradually reduced without the assistance of CD4^+^ T cells [4, 5]. CD4^+^ T cells are known to participate in all immune responses and have multiple effects. CD4^+^ T cells can differentiate into Th1, Th2, Th17 and regulatory T (Treg) cells. These cells mediate signals through cell-to-cell contact or cytokine secretion. Th1 cells mainly secrete cytokines such as IL-2, IFN-γ and TNF-α to eradicate viruses and parasites causing intracellular infections and play an important role in cellular immunity. Th2 cells participate in humoral immunity by secreting cytokines that can promote antibody production, such as IL-3, IL-4, IL-6 and IL-10. Then, the antibodies clear pathogens causing extracellular infections [6]. Treg cells (CD4^+^CD25^+^Foxp3^+^ regulatory T cells) are a group of CD4^+^ T cells with immunomodulatory effects that have a powerful immunosuppressive function. Foxp3 is a critical factor that can serve as a promotion factor for Treg cells. The main function of Foxp3 is regulating and maintaining Treg differentiation and development [7, 8]. Treg cells can reduce the effectiveness of HBV-specific T cell responses when the virus persists. Removal of Treg cells from patients can lead to HBV-specific T cell expansion and IFN-γ overproduction [9]. Therefore, CD4^+^ T cells play an important role in viral clearance and disease pathogenesis during HBV infection.

The weak T cell response of chronic hepatitis B patients (CHB) is associated with persistently high viral replication [2, 10]. Recent studies revealed that the sustained combination of exposure to antigens with high viral loads and excessive inhibitory signals in the liver microenvironment could lead to a progressive loss of T cell function and exhaustion of HBV-specific T cells [1, 3]. These “exhausted T cells” presented a state dysfunction of T cell and was firstly observed during chronic lymphocytic choriomeningitis virus (LCMV) infection in mice [11, 12] exhibit increased expression of immune inhibitory molecules, including programmed death-1 (PD-1), lymphocyte activation gene-3 (LAG-3 or CD223), T cell immunoglobulin domain and mucin domain 3 (TIM-3), CD244 (2B4) and CD160 [13–16]. Indeed, in our previous studies, we demonstrated high PD-1 and LAG-3 expression levels on exhausted CD8^+^ T cells during chronic HBV infection [17, 18].

PD-1 and LAG-3, which have been identified as markers of exhausted T cells in chronic diseases, play a role in homeostasis maintenance and immune regulation, especially during chronic viral infections resulting in depletion of T lymphocytes [18, 19]. Those inhibitory molecules have been associated with a hierarchical dysfunction of CD8^+^ T cell proliferation, cytokine production, and increased apoptosis [20–22]. However, the detailed roles of PD-1, LAG-3 and other inhibitory receptors in the development and maintenance of HBV CD4^+^ T cell dysfunction has not been elucidated.

In this study, we investigated the relationship between the expression of inhibitory molecules on CD4^+^ T cells in the peripheral blood and CHB disease progression. Furthermore, we sought to understand the functional impact of inhibitory molecules, such as PD-1 and LAG-3, as measured by changes in CD4^+^ T cell proliferation and IFN-γ, IL-2, TNF-α and IL-10 secretion.

## Results

### Expression of inhibitory receptors on the surface of peripheral blood CD4^+^ T cells from chronic hepatitis B patients

**Fig. 1:**
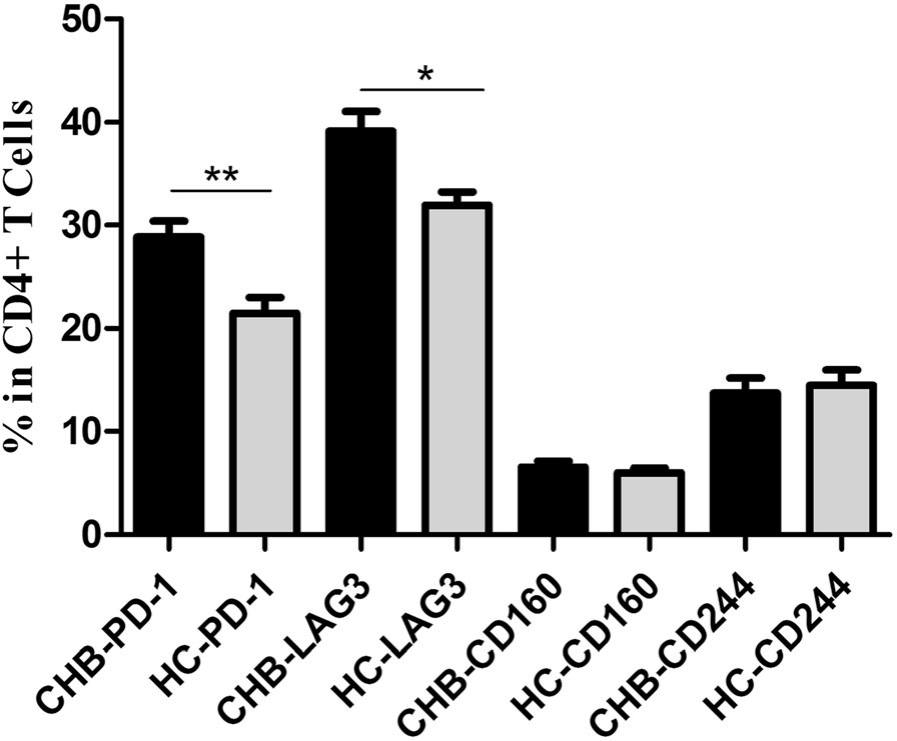
Distribution frequencies of inhibitory receptors on the surfaces of CD4^+^ T cells from the CHB patients and healthy individuals. Analysis of the percentages of PD-1^+^, LAG-3^+^, CD160^+^ and CD244^+^ CD4^+^ T cells from the HCs (*n* = 60) and CHB patients.*P < 0.05, **P < 0.01

The frequencies of CD4^+^ T cells with surface expression of the inhibitory receptors PD-1, LAG-3, CD160 and CD244 were evaluated in the CHB patient group (CHB group) and healthy control group (HC group) using flow cytometry. Significantly higher frequencies of PD-1^+^CD4^+^ and LAG-3^+^CD4^+^ cells were observed in the CHB group than in the HC group (*P* = 0.0014 and *P* = 0.0104, respectively). No difference was found in the CD160 and CD244 expression levels (Fig. 1).

### Correlation between hepatic injury and the PD-1 and LAG-3 expression levels on T cells

**Fig. 2:**
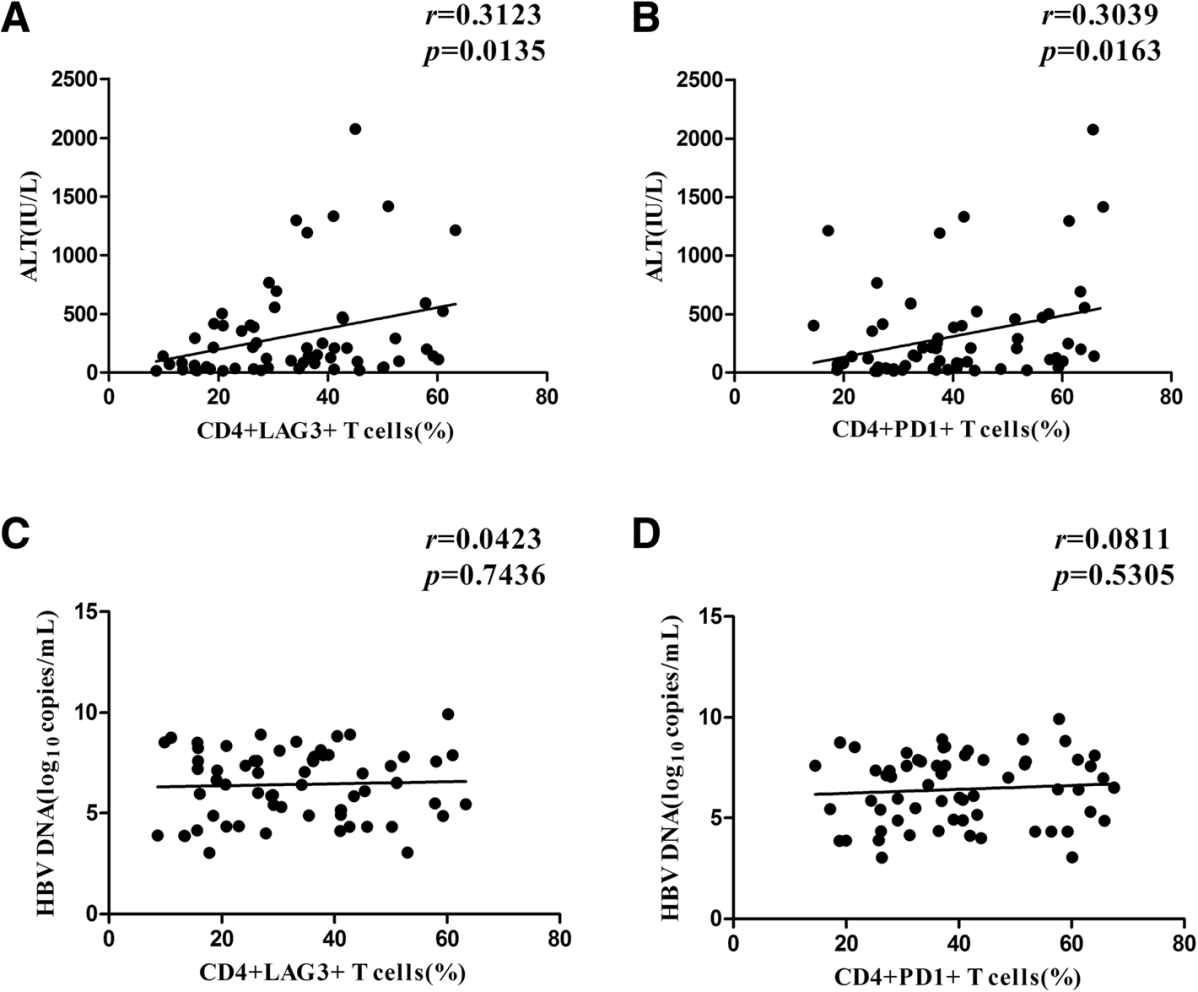
Association between the frequency of PD-1^+^and LAG-3^+^ CD4^+^ T cells and conventional markers for liver damage in CHB patients. **a**: Relationship between the serum ALT levels and the percentages of CD4^+^ LAG-3^+^ T cells in the CHB patients. **b**: The relationship between the serum ALT levels and the percentage of CD4^+^ PD-1^+^ T cells in the CHB patients. **c**: The relationship between the HBV DNA levels and the percentages of CD4^+^LAG-3^+^ T cells in the CHB patients. **d**: The relationship between the HBV DNA levels and the percentages of CD4^+^PD-1^+^ T cells in the CHB patients

Since PD-1 and LAG-3 were highly expressed by CD4^+^ T cells relative to the expression levels in the HC group, we analyzed the association between the PD-1 and LAG-3 expression levels on CD4^+^ T cells and the serum ALT levels (as a marker of hepatic injury) together with the HBV DNA levels among the HBV patients. A positive correlation was observed between PD-1 and LAG-3 expression and the serum ALT level (LAG3^+^CD4^+^: *r* = 0.3132, *P* = 0.0135, PD-1^+^CD4^+^: *r* = 0.3039, *P* = 0.0163, Fig. 2a, b). However, no association was found between the PD-1 and LAG-3 expression and HBV DNA levels (LAG3^+^CD4^+^: *r* = 0.0423, *P* = 0.7436, PD-1^+^CD4^+^: *r* = 0.0811, *P* = 0.5305, Fig. 2 c, d). These results suggested that the inhibitory receptors PD-1 and LAG-3 were highly expressed by CD4^+^ T cells from the CHB patients and were related to the degree of hepatic injury.

### Difference in cytokine production between the exhausted and non-exhausted CD4^+^ T cells

**Fig. 3:**
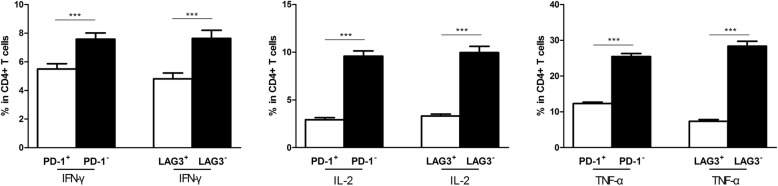
Comparison of Th1 cytokine levels in PD1^−^ and LAG-3^−^CD4^+^ T cells and in PD1^+^ and LAG-3^+^ CD4^+^ T cells. The proportions of IFN-γ, IL-2 and TNF-α-producing PD-1^−^ LAG-3^−^CD4^+^ and PD-1^+^LAG-3^+^CD4^+^ T cells. ***P < 0.001

The frequencies of CD4^+^ T cells with Th1 cytokine expression, such as IFN-γ, IL-2 and TNF-α, were assessed in the CHB group by flow cytometry. Higher IFN-γ, IL-2 and TNF-α expression levels were detected in both the PD1^−^CD4^+^ and LAG-3^−^CD4^+^ cells than in the PD1^+^CD4^+^ and LAG-3^+^CD4^+^ cells obtained from the CHB patients (all *P* < 0.0001, Fig. 3). This result demonstrated that high PD-1 and LAG-3 expression could lead to CD4^+^ T cell dysfunction.

### PD-L1 and LAG-3 antibodies improve the ability of Th1 cells to produce cytokines

**Fig. 4:**
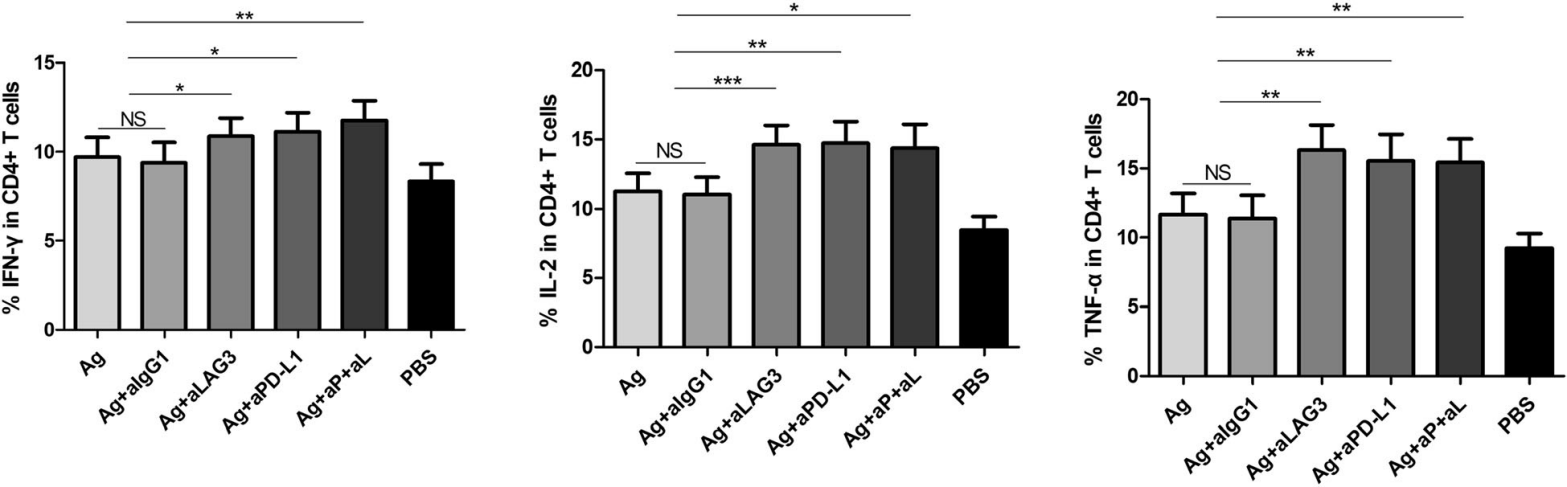
Effect of LAG-3 and PD-L1 blockade on Th1 cytokine release. IFN-γ, IL-2 and TNF-α expression on CD4^+^ T cells after HBV antigen stimulation with anti-IgG1 (isotype), anti-LAG-3, anti-PD-1 and anti-LAG-3 together with anti-PD-1 for 48 h. *P < 0.05, **P < 0.01, ***P < 0.001

Next, we characterized the reactivation of CD4^+^ T cells based on the production of Th1 cytokines, such as IFN-γ, IL-2 and TNF-α, following PD-L1 and LAG-3 blockade. IFN-γ, IL-2 and TNF-α production was significantly higher in the cells isolated from CHB patients and stimulated with HBcAg + anti-LAG-3, HBcAg + PD-L1 and HBcAg + anti-PD-L1 + anti-LAG-3 than in those stimulated with HBcAg alone (*P* = 0.019, *P* = 0.041 and *P* = 0.003; *P* = 0.0004, *P* = 0.003 and *P* = 0.014; and *P* = 0.002, *P* = 0.001 and *P* = 0.006, respectively). No significant differences were noted in cytokine production between the cells stimulated with HBcAg + IgG1 and HBcAg (Fig. 4). These results indicated that neither PD-L1 nor LAG-3 blockade was able to reactivate CD4^+^ T cell functions when compared to the ability of antigenic stimulation alone.

### The PD-L1 and LAG-3 antibodies suppress Foxp3 expression

**Fig. 5:**
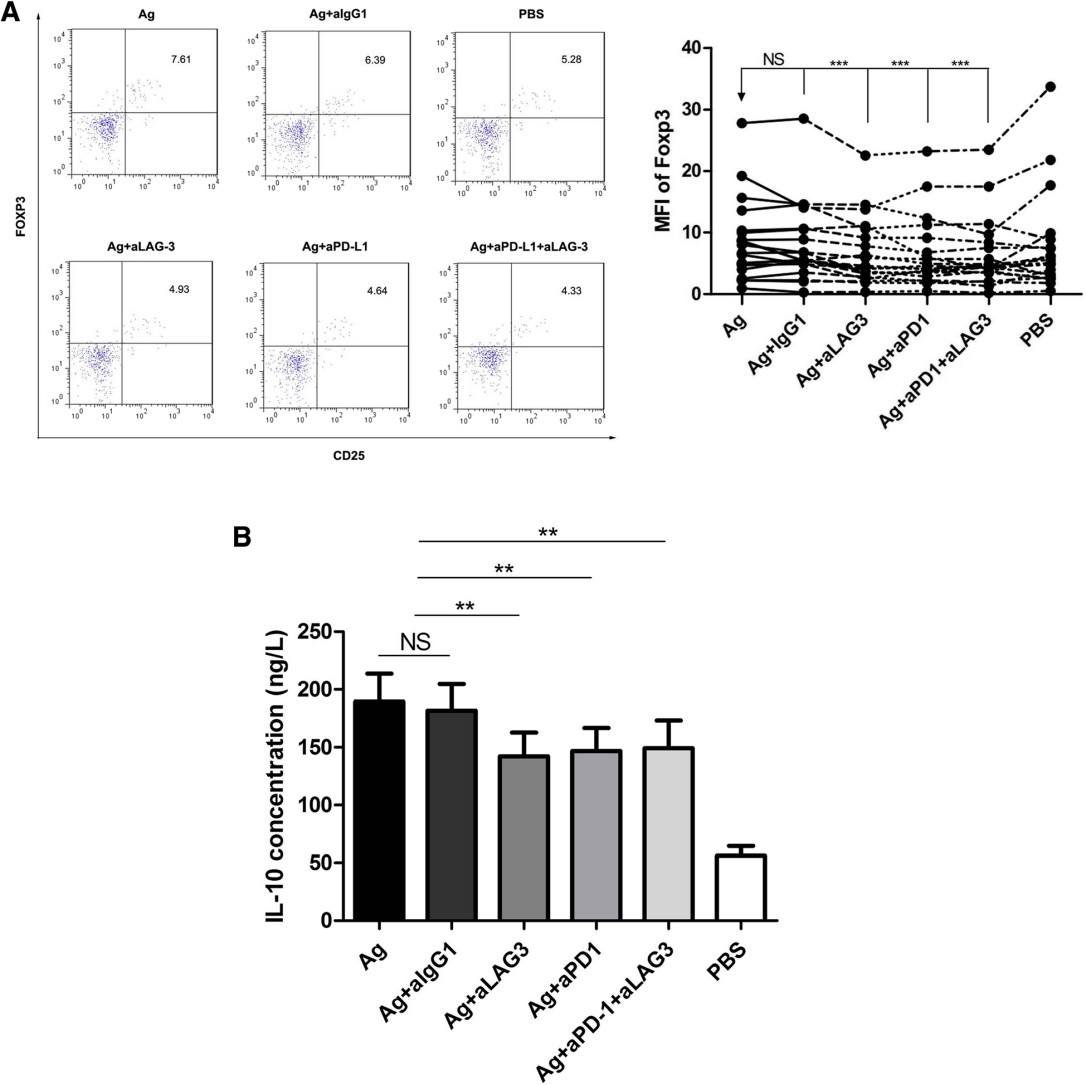
Effect of LAG-3 and PD-L1 blockade on Treg expansion and inhibitory cytokine secretion by CD4^+^ T cells. **a**. Foxp3 expression on CD25^+^ T cells after HBV antigen stimulation with anti-IgG1 (isotype), anti-LAG-3, anti-PD-1, and anti-LAG-3 with anti-PD-1 for 48 h. Graphs showing events after gating on CD3^+^CD4^+^ T cells. **b** IL-10 secretion from CD4^+^ T cells after incubation of antigens with anti-IgG1 (isotype), anti-LAG-3, anti-PD-1 and anti-LAG-3 together with anti-PD-1 for 48 h. **P < 0.01, ***P < 0.001

Treg surface markers, including CD4, CD25 and Foxp3, were analyzed by flow cytometry. The proportion of Foxp3^+^ cells among the CD4^+^CD25^+^ cells was lower when the cells were stimulated with HBcAg + anti-LAG-3, HBcAg + PD-L1 and HBcAg + PD-L1 + anti-LAG-3 than when stimulated they were with HBcAg alone or HBcAg + IgG1 (*P* = 0.0005, *P* < 0.0001 and *P* = 0.0002, respectively; Fig. 5a). These findings suggested that blocking PD-L1 or LAG-3 would prevent the production of Tregs.

### The PD-L1 and LAG-3 antibodies reduced IL-10 secretion from CD4^+^ T cells

We used ELISA to measure the cytokine levels in the culture supernatants from CD4^+^ T cells stimulated with HBcAg, HBcAg + IgG1, HBcAg + anti-LAG-3, HBcAg + anti-PD-L1 and HBcAg + PD-L1 + anti-LAG-3. We found that IL-10 could be inhibited by the LAG-3 or PD-L1 antibody (*P* = 0.002 *P* = 0.005 and *P* = 0.004, respectively, compared with HBcAg alone; Fig. 5b). We speculated that PD-L1 and LAG-3 blockade contributed to the reduced inhibition of CD4^+^ T cells. Other cytokines, including IL-6, IL-4 and TGF-β, were also examined. However, no change in IL-6 was found, and the IL4 and TGF-β levels were too low to be detected (data not shown).

## Discussion

T cell exhaustion was first described 20 years ago by Zajac and Gallimore in a lymphocytic choriomeningitis virus (LCMV)-infected mice suffering from specific CD8^+^ T cell dysfunction [11, 12]. A similar phenomenon was later confirmed in HBV, HCV, and cancer patients [23]. Researchers discovered that T cells exhibited progressive and gradual exhaustion during persistent infection [24, 25]. T cell exhaustion begins once the virus begins to replicate on a massive scale, as shown by higher expression of inhibitory molecules (such as PD-1 and LAG-3) and limited T cell proliferation and dysfunction [26, 27]. Eventually, T cell exhaustion results in inhibition of host immune responses and hence the pathogen becomes dominant, leading to persistent infection.

T cell exhaustion is a status of gradual T cell dysfunction that arises during chronic infections. The inhibitory receptors PD-1 and LAG-3 are expressed successively on the cell surface and emerge only when the cells are close to apoptosis. The numbers and types of receptors are closely related to the degree of T cell exhaustion [28, 29]. During HBV infection and clearance, CD4^+^ T cells are the key factor regulating on the cellular CTL response to HBV [30–32]. According to previous research, the lack of CD4^+^ Th cells was the main cause of CD8^+^ T cell exhaustion [33]. Although CD4^+^ T cells have remained important for T cell exhaustion [34, 35], the mechanism of CD4^+^ T cell exhaustion in chronic HBV-infected patients is not well understood. Mueller et al. reported that efficient presentation of durable virus antigen resulted in T cell exhaustion [36]. High expression of inhibitory receptors on CD8^+^ T cells was related to sustained viral recognition [22]. The amount and variety of inhibitory receptors increased during chronic HBV infection, and infection led to T cell dysfunction, deviation from normal effector cells, and apoptosis [22, 37, 38]. Previous research had shown that blocking PD-1 or LAG-3 pathway can stimulate T-cell activation and proliferation to improve immunity and clearance of tumors and virus [21, 39].

In this study, we compared the distribution frequencies of both PD-1 and LAG-3 on CD4^+^ T cells from CHB patients and healthy individuals. The distribution frequencies of PD-1 and LAG-3 on CD4^+^ T cells from CHB patients were significantly higher than those from healthy individuals. Furthermore, our results showed a significant positive correlation between PD-1 and LAG-3 expression on CD4^+^ T cells with conventional markers for hepatic injury, such as ALT. This result indicated that the distribution frequencies of PD-1 and LAG-3 were positively correlated with the level of liver inflammation.

Tregs are a type of T cell subset that encompasses a large population of lymphocytes. These cells play pivotal roles in maintaining immune homeostasis, have immunosuppressive functions and can inhibit the activation and proliferation of CD4^+^ and CD8^+^ T cells by secreting inhibitory cytokines [40]. In patients with chronic HBV infection, local expression of co-inhibitory receptors and immunosuppressive mediators results in a unique immune regulatory environment in the liver with dysfunctional T cells. This hepatic suppressive microenvironment consists primarily of higher numbers of Tregs, upregulated programmed death-1/programmed death ligand-1 (PD-1/PD-L1) signals and low levels of Toll-like receptor (TLR) expression [2, 41]. Previous research indicated that chronic HBV infection was related to an increase in Tregs and defective CD8^+^ T cells that failed to produce IFN-γ [42, 43]. Help from CD4^+^ T cells is important for maintenance of CD8^+^ T cell function during chronic infections, but CD4^+^ T cells also lose this capacity during chronic HBV infections [44]. In our study, PD-1 and LAG-3 blockade partially inhibited CD4^+^CD25^+^Foxp3^+^ Treg expansion and suppressed inhibitory cytokine IL-10 secretion from CD4^+^ T cells. We also observed that the ability of CD4^+^ T cells to produce IFN-γ, IL-2, and TNF-α was improved by blocking PD-1 and LAG-3. This result indicated that CD4^+^ T cell functions could be partly recovered by PD-1 and LAG-3 blockade.

T cell functions can be regulated by multiple inhibitory molecules, including PD-1, LAG-3, CD224, CD160, T cell immunoglobulin mucin-3 (Tim-3) and CTLA-4 [10, 16]. A recent study detected high LAG-3 and PD-1 expression levels in chronic LCMV infections, which could lead to CD8^+^ T cell dysfunction [45, 46]. However, very little research into CD4^+^ T cell exhaustion is available. Our current study revealed that the PD-1 and LAG-3 expression levels regulated the functions of CD4^+^ T cells during chronic HBV infection. CD4^+^ T cells with PD-1 and LAG-3 expression exhibited reduced IFN-γ, IL-2 and TNF-α production. However, CD4^+^ T cell function was restored when PD-1 and LAG-3 activity was inhibited by treatment with PD-L1 and LAG-3 antibodies.

Our findings characterize the intricate mechanisms that regulate the immune response during chronic HBV infection and may have therapeutic implications for future T cell function therapies.

## Conclusion

In summary, during HBV infection, CD4^+^ T cells appeared as high expression of PD-1 and LAG-3 but loss of helper T cells’ fuction such as decreased secretion of IFN-γ, IL-2 and TNF-α. After blocking PD-L1 and LAG-3, the function of CD4^+^ T cells in chronic hepatitis B patients can be partially restored. Our findings could provide a new therapeutic implications for future T-cell function therapies.

## Methods

### Patients

**Fig. 6:**
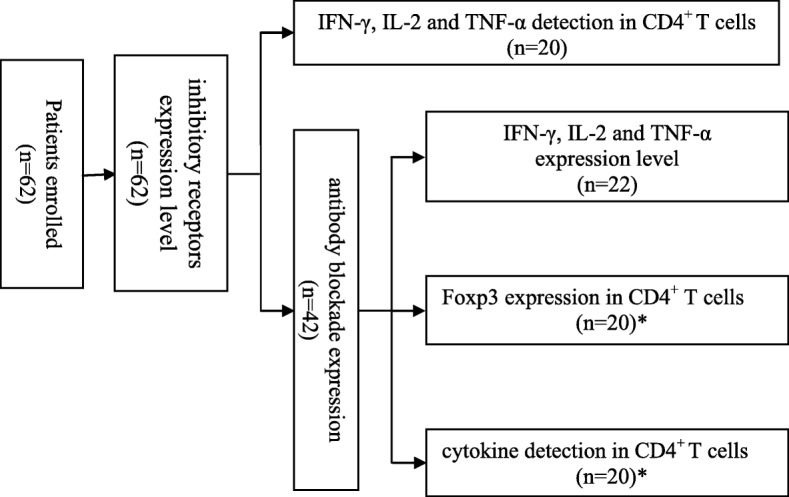
Flowchart of patients for each experiment in this study. *these patients were used for both Foxp3 and cytokine detection

A total of 62 treatment-naïve active CHB patients were involved in this study, and 60 healthy individuals served as controls (HCs) in parallel. The details for each experiment for these patients (such as inhibitory molecule expression levels in CD4^+^ T cells and the effect of blocking antibodies on CD4^+^ T cells) are provided in Fig. 6. The diagnostic criteria for CHB referred to the American Association for the Study of Liver Diseases (AASLD) Practice Guidelines [47].

The clinical characteristics of all study groups are presented in Table 1. Patients with autoimmune disease, diabetes, hyperthyroidism, hematological system diseases, and other hepatotropic disease were excluded from the study. Patients who received HBV treatment within 6 months prior to blood sampling were also excluded. Written informed consent was obtained from all individuals according to the Declaration of Helsinki (1964). The Medical Ethics Committee of the First Affiliated Hospital, School of Medicine, Zhejiang University (Hangzhou, China), approved the study.

**Table 1:** Clinical characteristics of the studied groups

Variables	CHB	HC
Age	33.77 ± 1.09	35.45 ± 1.58
Gender (M/F)	62 (48/14)	60 (44/16)
HBeAg (+/−)	39/23	0/60
ALT (IU/L)	313.7 ± 53.31	20.88 ± 0.81
HBV-DNA (copies/mL)	> 5.00 × 10^3^ – 8.46 × 10^9^	< 5.00 × 10^3^

Note: Data are presented as mean ± SEM. *M/F* male/female, *HBeAg (****+/−****)* HBeAg-positive and negative, *CHB* chronic hepatitis B patients, *HC* healthy control

### Analysis of serum HBV markers and liver function

Serum ALT was measured using automated biochemical techniques (Hitachi 7600, Tokyo, Japan) (upper limit of normal: 35 IU/L). The serum HBeAg level was determined using the Chemiluminescent Microparticle Immunoassay (CMIA) kit for the Architect-i2000 system (Abbott Laboratories, Chicago, IL, USA), with a positive result recorded as S/CO ≥ 1.0. The serum HBV DNA load was also determined by ABI 7300 fluorescent quantitative PCR (Applied Biosystems Corporation, Foster City, CA, USA), with a detection limit of 300 viral genome copies/mL.

### Peripheral blood mononuclear cell isolation

Peripheral blood mononuclear cells (PBMCs) were isolated from blood samples by Ficoll-Hypaque density gradient centrifugation (Amersham Pharmacia, Uppsala, Sweden). The growth medium was supplemented with 10% heat-inactivated fetal calf serum (GIBCO, USA), 100 units/mL of penicillin and 100 μg/mL of streptomycin, and the cells were cultured at 37 °C with 5% CO_2_.

### Flow cytometry analysis

The PBMCs were resuspended in PBS buffer and then incubated with anti-CD4-FITC (Becton Dickinson Biosciences, USA), anti-CD223-APC (R&D Systems, Inc., USA.), anti-PD-1-PE-Cy7 (BioLegend, USA), anti-CD160-PE (BioLegend, USA) and anti-CD244-PerCP-Cy5.5 (BioLegend, USA) antibodies at room temperature for 30 min in the dark. Immunoglobulin IgG isotype-matched antibodies served as the negative controls. The stained cells were analyzed using the FACScan™ system (Becton Dickinson Biosciences, USA).

### Isolation and stimulation of CD4^+^ T cells

CD4^+^ T cells were enriched from PBMCs by positive selection using magnetic-activated cell-sorting columns (Miltenyi Biotec, Germany) and adjusted to a cell density of ~ 1 × 10^6^ cells/mL. Purified CD4^+^ T cells were stimulated for 72 h at 37 °C with HBV core antigen (1 μg/mL; Meridian, BioDesign, USA) + PBS (control; GIBCO, USA), HBV core antigen (1 μg/mL; Meridian, BioDesign, USA) + anti-IgG1 (1 μg/mL; eBioscience, USA), HBV core antigen + anti-PDL1 (1 μg/mL; eBioscience, USA), HBV core antigen + anti-LAG-3 antibody (1 μg/mL; Abcam, UK), and HBV core antigen + anti-PDL1 (1 μg/mL) + anti-LAG-3 antibody (1 μg/mL). Subsequently, the cell culture supernatants were collected and stored at − 80 °C for ELISA, and the cells were collected for flow cytometry.

### Determination of intracelluar cytokine release by flow cytometry

After 72 h of in vitro stimulation, the cells were incubated with a cell stimulation cocktail (1:500, eBioscience, USA). After 5 h of incubation, the cells were stained with anti-CD4-APC (BioLegend, USA) at room temperature for 30 min in the dark. After fixation and permeabilization, the cells were stained with anti-IFN-γ-PerCP-Cy5.5 (BioLegend, USA), anti-IL-2-PE (BioLegend, USA), and anti-TNF-α-FITC (BioLegend, USA) at room temperature for 30 min in the dark. Immunoglobulin IgG isotype-matched antibodies served as the negative controls. The cells were analyzed with the FACScan system.

### Determination of Foxp3 expression by flow cytometry

To detect Foxp3, CD4^+^ T cells were incubated with anti-CD4-FITC and anti-CD25-APC (eBioscience, USA). After permeabilization and fixation, the cells were incubated with anti-Foxp3-PE or an IgG1 control (eBioscience, USA) at room temperature for 30 min in the dark. Then, the cells were then analyzed with the FACScan system.

### Cytokine detection by ELISA

Sandwich ELISA technology was used to measure the concentrations of human IL-10, TGF-β and IL-4 in the CD4^+^ T cells. All Quantikine ELISA kits (BioLegend, USA) were used according to the manufacturer’s instructions.

### Statistical analysis

Continuous variables are presented as the mean ± standard error of the mean (SEM). The Mann-Whitney U test was used to compare the HBV group with the healthy control group, and the Wilcoxon signed rank test was used to analyze differences between the anti-PDL1/LAG-3-treated and untreated groups. The correlations between the PD-1 and LAG-3 expression levels and the HBV DNA and ALT levels were analyzed by Pearson’s correlation analysis. The data were analyzed using GraphPad Prism 7.0. *P* values < 0.05 were considered statistically significant.

## Data Availability

Since the data has not yet been used in a patent application, the data will not be shared.

## Abbreviations

ALT: Alanine aminotransferase
CHB: Chronic hepatitis B
Foxp3: Forkhead box P3
HBV: Hepatitis B virus
HC: Healthy controls
IFN-γ: Interferon γ
IL-2: Interleukin-2
LAG-3: Lymphocyte activation gene-3
PBMC: Peripheral blood mononuclear cells
PD-1: Programmed death-1
TNF-α: Tumor necrosis factor α
